# Glucose‐lowering effects of physical activity in type 1 diabetes: A causal modelling and matched‐pair analysis approach

**DOI:** 10.1111/dme.70146

**Published:** 2025-10-08

**Authors:** John S. Pemberton, Catherine L. Russon, Richard M. Pulsford, Brad S. Metcalf, Emma Cockcroft, Michael J. Allen, Anne M. Frohock, Rob C. Andrews

**Affiliations:** ^1^ Birmingham Women's and Children's Hospitals NHS Foundation Trust Diabetes Centre Birmingham UK; ^2^ University of Exeter Medical School Exeter UK; ^3^ Oxford University Hospitals NHS Foundation Trust Oxford UK; ^4^ NIHR Exeter Clinical Research Facility Exeter UK

**Keywords:** Diabetes, endocrine, exercise

## Abstract

**Aims:**

To evaluate the acute glucose‐lowering effect of bouts of physical activity (PA) for hyperglycaemia in individuals with type 1 diabetes, using a within‐subject matched‐pairs causal design to approximate the control condition of no activity.

**Methods:**

Data comprised 1546 PA bouts of 10–30 min from 482 participants in the T1DEXI and T1DEXIP cohorts where glucose was >10 mmol/L. Each PA bout was matched [starting glucose, glucose rate of change, insulin on board (IOB) and glucose variability (CV)] to a matched non‐PA period within the same individual using a weighted k‐nearest neighbours algorithm (SMD <0.01). Primary outcome: Change in glucose from PA onset to 20 min post‐activity. Secondary outcomes: Predictors of glucose response and rate of hypoglycaemia incidence.

**Results:**

PA [median 23 min: IQR (20, 30)] led to a mean glucose change of −2.2 mmol/L (*p* < 0.001), compared with 0.3 mmol/L (*p* < 0.001) during matched non‐PA periods (mean difference: −1.9 mmol/L (*p* < 0.0001)). No significant differences by age, activity type or intensity were observed. The strongest predictors of PA‐induced glucose change were (in order) glucose rate of change, starting glucose, CV, duration and IOB. A heatmap using starting glucose and glucose rate of change was developed to guide real‐time decision‐making. PA‐induced hypoglycaemia risk was very low (<2%).

**Conclusion/Interpretation:**

Using PA to lower high glucose levels is an effective and safe strategy, and when guided by CGM, it can become a personalised tool for type 1 diabetes education.

## Graphical abstract

In people with type 1 diabetes, 10–30 min of physical activity lowers glucose by 2.2 mmol/L on average and is eight times more effective than matched non‐activity periods. This real‐world analysis supports the use of CGM data to personalise and time activity for optimal glucose management.

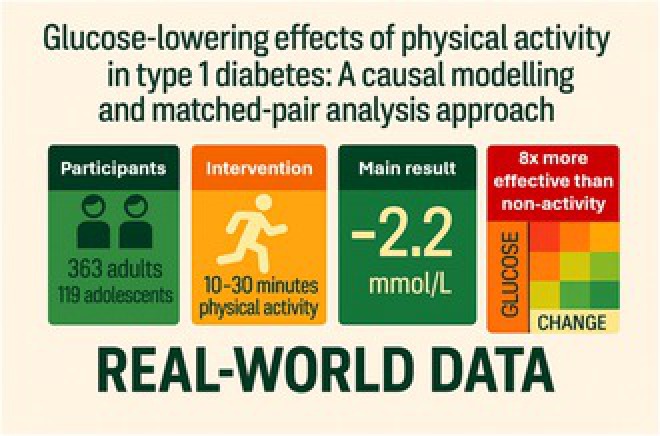

**What's new?**

**What is already known?**

- Physical activity is recommended for glucose management in type 1 diabetes, but guidance on how to use it acutely during hyperglycaemia is lacking.

**What did the study find?**

- In a within‐subject matched‐pair analysis of 1546 activity bouts, 23 min of physical activity reduced glucose by 2.2 mmol/L on average—eight times greater than matched rest periods.

**What are the implications of the study?**

- This study provides real‐world evidence supporting the use of physical activity, alongside insulin therapy, as a safe and effective strategy for managing hyperglycaemia in people with type 1 diabetes.

## 1 INTRODUCTION

Achieving optimal glycaemic control in type 1 diabetes remains a significant challenge. Despite widespread use of continuous glucose monitoring (CGM) and hybrid closed‐loop (HCL) systems, most users fail to reach the recommended target of ≥70% time in range (TIR; 3.9–10 mmol/L).^1^, ^2^, ^3^ Hyperglycaemia is usually corrected with rapid‐acting insulin, which can have variable onset and duration of action, especially in the context of recent meals or activity, leading to inconsistent glucose responses.^4^ This highlights the urgent need for complementary strategies to manage hyperglycaemia.

Physical activity (PA) can serve as a non‐pharmacologic strategy for hyperglycaemia management, lowering glucose through insulin‐independent mechanisms and enhancing insulin sensitivity,^5^, ^6^ with experimental data demonstrating significant reductions in glucose.^7^ Observational real‐world studies provide some evidence of efficacy in lowering glucose levels above 10 mmol/L,^8^ but the absence of counterfactual comparators in these studies limits causal inference and undermines the application of PA as an acute glucose‐lowering intervention.

Causal modelling offers a reliable way to estimate the effects of different factors in observational data by mimicking the conditions of a randomised controlled trial.^9^ Applying causal modelling to real‐world PA events, while accounting for both individual and event‐specific factors, can generate robust evidence regarding the impact of everyday behaviours, supporting the development of new evidence‐based interventions.

This study employed a novel within‐subject matched‐pairs approach applied to the T1DEXI^10^ and T1DEXIP^11^ datasets, intending to provide the first causal evidence for the glucose‐lowering effect of free‐living PA in individuals with type 1 diabetes.

The primary aim was to compare the change in glucose concentration from activity onset to 20 min post‐activity for 10‐ to 30‐min bouts of PA initiated at glucose levels >10 mmol/L. The secondary aims were to identify moderators of glucose responses between PA and matched non‐PA periods and to assess the incidence of hypoglycaemia for the same period compared with matched non‐PA periods under free‐living conditions. Finally, to develop a user‐friendly tool to help individuals with type 1 diabetes understand the likely glucose‐lowering effect of physical activity.

## 2 METHODS

**FIGURE 1:**
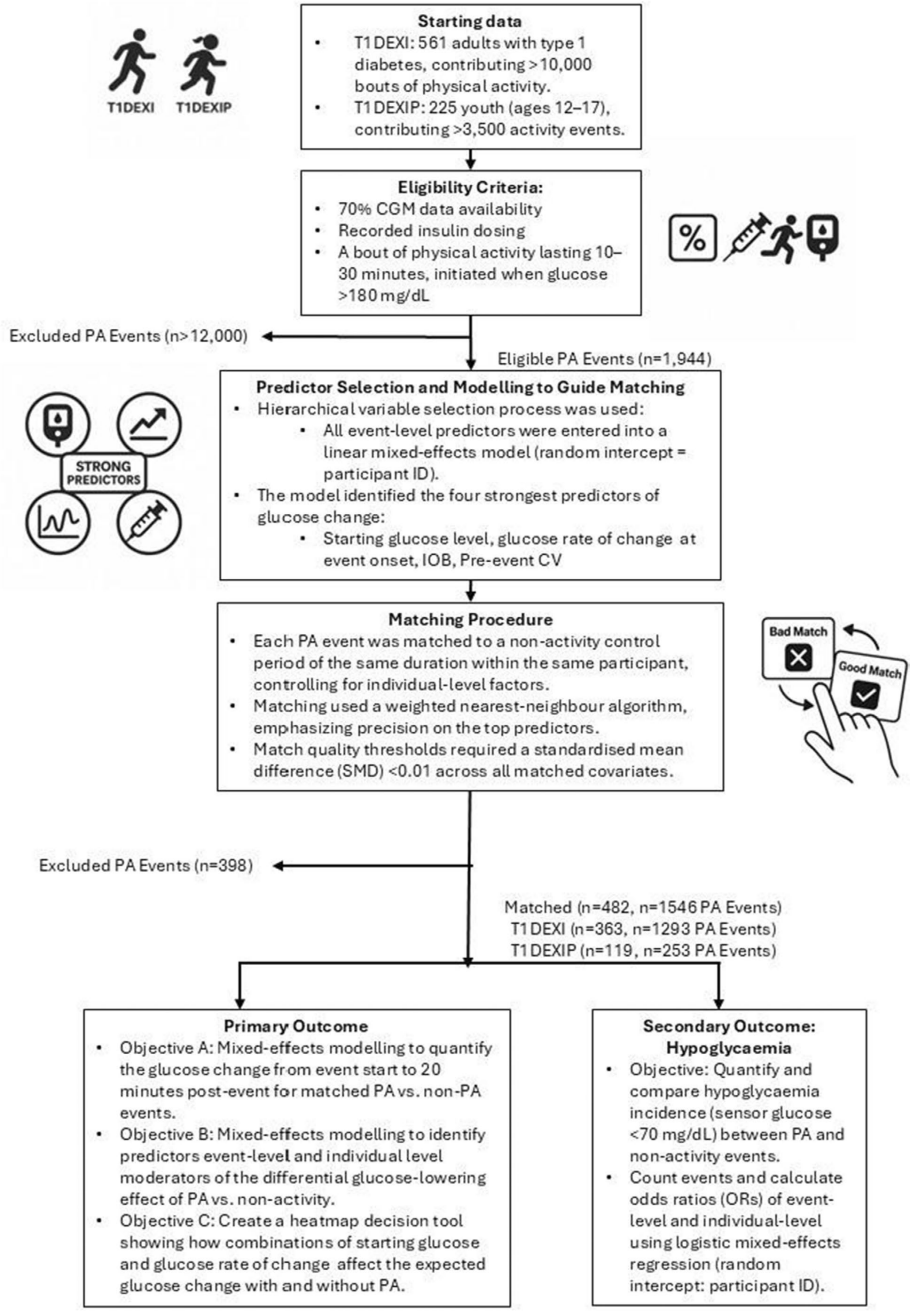
Overview of the analysis plan using matched‐pairs design. This flowchart outlines the analytical workflow for evaluating the glucose‐lowering effect of physical activity (PA) in type 1 diabetes (T1D) using a within‐subject matched‐pairs design. PA refers to physical activity, and T1D stands for type 1 diabetes. T1DEXI and T1DEXIP refer to the type 1 diabetes exercise initiative adult and paediatric cohorts, respectively. IOB represents insulin on board at the start of the event. CV is the coefficient of variation of glucose in the hour prior to activity, and SMD refers to the standardised mean difference used to assess the quality of matching between PA and non‐PA.

An overview of the methods used in this study is available in Figure 1.

### 2.1 Study Design and Participants

This study was a retrospective analysis of two pre‐existing datasets. In T1DEXI (561 adults, >10,000 PA bouts), PA was captured using accelerometry, while in T1DEXIP (225 adolescents aged 12–17 years, >3500 PA bouts), PA was self reported via daily logs. Insulin and carbohydrate intake were recorded via pump downloads, injection logs and participant self report, as per original study protocols.^8^, ^12^ All participants were using continuous glucose monitoring and insulin therapy in free‐living conditions. Eligibility for this analysis required 70% CGM data availability, insulin dosing data, with at least one qualifying bout of PA lasting 10–30 min and initiated at glucose >10 mmol/L. PA bouts were identified via accelerometer (T1DEXI) or self report (T1DEXIP) and included if they lasted between 10 and 30 min. Duration was self‐selected in T1DEXIP and for most T1DEXI participants, reflecting real‐world constraints. However, adult participants were prescribed six 20‐min PA sessions over 30 days, as part of the original T1DEXI protocol.^8^, ^12^ Only PA bouts with sufficiently matched non‐PA periods were included to allow for robust within‐subject comparisons. Full cohort characteristics and data collection methods are detailed in the original publications.^10^, ^11^

Starting glucose was defined as the CGM reading at PA onset (time zero). Glucose rate of change (ROC) was calculated as the mean of the three 5‐min CGM ROC values immediately prior to PA onset. Insulin on board (IOB) was estimated from insulin pump records or injection logs using standard decay models for rapid‐acting insulin analogues over a 4‐h window, consistent with prior work.^8^

### 2.2 Sample Size Justification

This study leveraged the full available dataset of eligible PA events from the T1DEXI and T1DEXIP cohorts. Of 1943 candidate PA bouts starting above 10.0 mmol/L, 1546 were successfully matched to non‐PA bouts from the same individuals using a strict covariate balance threshold (standardised mean difference <0.1 across matching variables). No formal power calculation was conducted a priori due to the retrospective nature of the dataset; however, the achieved sample size exceeded that of most prior real‐world studies evaluating glucose response to physical activity in type 1 diabetes. Further methodological detail via a STROBE checklist^13^ is reported in Table S7.

### 2.3 Outcomes

The primary outcome was the change in sensor glucose from the start of PA to 20 min post‐activity, accounting for CGM lag,^12^ comparing 10‐ to 30‐min PA bouts to matched non‐PA periods. All bouts began at a glucose level >10 mmol/L under free‐living conditions. The secondary outcome was assessing hypoglycaemia incidence (<3.9 mmol/L) during or within 20 min after PA, compared with matched control periods.

### 2.4 Causal Matched‐Pairs Design

Rather than relying on propensity scores, which have well‐documented limitations for causal inference in matched designs,^14^ we adopted an outcome‐oriented approach. Specifically, we prioritised matching on covariates that most strongly influenced glucose change following PA.

To identify covariates for matching, we conducted a variable selection process. Variables significantly associated with glucose change in univariate Pearson correlations were entered into a multivariable linear mixed‐effects model with a random intercept for participant ID. Variables retained in this model were used for matching.

Each PA bout was matched to a non‐PA control period of equal duration, drawn from the same participant's CGM data. Matched control periods could occur before or after the PA bout, with no systematic preference. Time of day was entered as a continuous variable (clock time) rather than categorised. This within‐subject design inherently controlled for baseline characteristics such as age, sex, insulin modality and HbA1c.

We used a k‐nearest neighbour (kNN) matching algorithm^15^ to pair PA bouts with non‐PA bouts from the same participant. This method was selected over inverse probability of treatment weighting and propensity score matching because it allowed direct matching on multiple continuous variables, producing tighter covariate balance without requiring categorisation or reduction to a single score. Matching variables were selected based on both theoretical relevance^10^, ^16^ and observed univariate associations, reducing the risk of overmatching. Callipers were calculated as 0.2 × SD of each variable's pooled matched distribution. Although our kNN algorithm was custom‐coded to incorporate variable weights from the multivariable model, similar procedures can be implemented in standard software such as the MatchIt package in R.

To minimise confounding, overnight periods and time windows within 1 h before or 4 h after PA bouts were excluded. A buffer of 40 min on either side was also applied to prevent overlap between matched periods.

Final match quality was assessed using standardised mean differences (SMDs), with values below 0.1 considered indicative of acceptable covariate balance.^17^

### 2.5 Statistical Analysis

Descriptive statistics were reported as the mean (±SD), median (IQR: 25th, 75th) or *n* (%), as appropriate. Glucose changes are reported as means with 95% confidence intervals (95% CI). Quadratic and cubic terms were tested alongside standard terms at every stage of analysis. Two‐sided *p*‐values <0.05 were considered statistically significant.

A three‐level linear mixed‐effects model was used to estimate the association between PA and glucose change. Each observation was nested within a matched PA bout/non‐PA period (Level 2), which was nested within participant (Level 3). Candidate variables identified from the literature were included in the mixed‐effects model, regardless of univariate correlation to avoid bias from univariate screening.^18^ The model included multiple periods per participant with a random intercept for participant ID. The model included fixed effects for PA condition and relevant covariates (see Data: S1–S3), as well as interaction terms to assess whether the PA effect on glucose changes varied by physiological state.

Hypoglycaemia risk was modelled using a logistic mixed‐effects regression with fixed effects for covariates and a random intercept for participants to account for within‐subject clustering. Odds ratios (ORs) were estimated to compare the likelihood of hypoglycaemia between activity conditions.

All analyses were performed in Python (v3.9) and R (v4.3.1), using the Diametrics,^19^ lme4,^20^ lmerTest, Pandas,^21^ NumPy and statsmodels packages.^22^ All code is available at https://github.com/cafoala/glucoseLo‐stats.

### 2.6 ETHICS STATEMENT

This study involved secondary analysis of de‐identified data from the T1DEXI and T1DEXIP studies, both of which obtained institutional review board approval and informed consent from all participants, as described in the original publications.^10^, ^11^

This secondary analysis was conducted in accordance with the principles of the Declaration of Helsinki.

## 3 RESULTS

### 3.1 Participant Characteristics

A total of 482 participants were included in the analysis, comprising 363 from the T1DEXI cohort and 119 from T1DEXIP. The T1DEXI group had a median age of 32 years (IQR: 25–45), compared with 13 years^8^, ^12^ in the T1DEXIP group. In the T1DEXI cohort, 44% of participants were using HCL systems, 41% were on standard insulin pumps and 15% were managing diabetes via MDI. For the T1DEXIP group, 56% were using HCL systems, with 29% on pumps and 15% using MDI. Full demographic details are available in Table 1.

**TABLE 1:** Participant characteristics, baseline glycaemic metrics and physical activity bout features in the overall cohort and by study group (T1DEXI vs. T1DEXIP).

	Overall	T1DEXI	T1DEXIP
Demographic characteristics			
Number of participants	482	363	119
Age	26 [18, 39]	32 [25, 45]	13 [12, 14]
BMI	24 [22, 27]	25 [23, 27]	21 [18, 24]
Insulin modality	Closed loop 226 (47%) Insulin pump 183 (38%) MDI 73 (15%)	Closed loop 160 (44%) Insulin pump 148 (41%) MDI 55 (15%)	Closed loop 66 (56%) Insulin pump 35 (29%) MDI 18 (15%)
HbA1c (mmol/mol)	51 [45, 56]	50 [44, 54]	54 [49, 62]
HbA1c (%)	6.8 [6.3, 7.3]	6.7 [6.2, 7.1]	7.1 [6.6, 7.8]
Years since diagnosis	12 [9, 20]	15 [10, 23]	10 [7, 11]
Race	American Indian/Alaskan Native: 2 (0.4%), Asian: 10 (2.1%), Black/African American: 8 (1.7%), Multiple: 11 (2.3%), Not reported: 14 (2.9%), Unknown: 2 (0.4%), White: 435 (90%)	American Indian/Alaskan Native: 2 (0.6%), Asian: 9 (2.5%), Black/African American: 7 (1.9%), Multiple: 6 (1.7%), Not reported: 10 (2.8%), Unknown: 1 (0.3%), White: 328 (90.4%)	American Indian/Alaskan Native: 0 (0.0%), Asian: 1 (0.8%), Black/African American: 1 (0.8%), Multiple: 5 (4.2%), Not reported: 4 (3.4%), Unknown: 1 (0.8%), White: 107 (89.9%)
Sex	Female: 329 (68%) Male: 153 (32%)	Female: 281 (77%) Male: 82 (23%)	Female: 48 (40%) Male: 71 (60%)
Glycaemic characteristics			
Average glucose (mmol/L)	8.4 [7.7, 9.5]	8.1 [7.5, 9.1]	9.4 [8.3, 10.4]
SD (mmol/L)	2.9 [2.4, 3.4]	2.7 [2.3, 3.3]	3.2 [2.7, 3.8]
CV (%)	34 [30, 37]	34 [30, 37]	34 [31, 38]
Time in range 3.9–10.0 mmol/L (%)	72 [58, 80]	73 [62, 82]	62 [51, 73]
Time below range <3.9 mmol/L (%)	2.1 [0.8, 3.7]	2.3 [1.1, 4.1]	1.0 [0.4, 2.5]
Time above range >10 mmol/L (%)	26 [16, 39]	23 [15, 34]	37 [24, 48]

Characteristic	Physical activity	Non‐activity	Physical activity	Non‐activity	Physical activity	Non‐activity
Event characteristics						
Number of bouts	1546	1546	1293	1293	253	253
Duration of bout (min)	24 [20, 30]	24 [20, 30]	25 [20, 30]	25 [20, 30]	20 [15, 30]	20 [15, 30]
Insulin on board (U/kg)	0.01 [0.00, 0.03]	0.02 [0.01, 0.03]	0.01 [0.00, 0.03]	0.02 [0.00, 0.03]	0.02 [0.00, 0.04]	0.02 [0.01, 0.05]
Starting glucose (mmol/L)	11.7 [10.7, 13.1]	11.6 [10.7, 13.0]	11.6 [10.7, 12.9]	11.5 [10.7, 12.9]	11.9 [10.8, 14.0]	12.0 [10.8, 13.9]
Starting rate of change (mmol/L/ min)	0.00 [−0.03, 0.04]	0.00 [−0.03, 0.04]	0.00 [−0.03, 0.04]	0.00 [−0.03, 0.04]	0.00 [−0.03, 0.04]	0.00 [−0.03, 0.04]
CV in the 1 h prior to PA (%)	5.9 [3.4, 9.6]	5.7 [3.5, 9.2]	5.9 [3.4, 9.9]	5.7 [3.4, 9.4]	5.6 [3.2, 8.7]	5.7 [3.7, 8.5]
Time of day	Morning: 390 (25%) Afternoon: 700 (45%) Evening: 456 (30%)	Morning: 467 (30%) Afternoon: 467 (30%), Evening: 619 (40%)	Morning: 335 (26%) Afternoon: 560 (43%), Evening: 398 (31%)	Morning: 373 (29%), Afternoon: 402 (31%), Evening: 518 (40%)	Morning: 55 (22%) Afternoon: 140 (55%) Evening: 58 (23%)	Morning: 94 (37%) Afternoon: 58 (23%) Evening: 101 (40%)
Type of PA	Aerobic: 875 (57%) Mixed: 537 (35%) Anaerobic: 134 (8%)	N/A	Aerobic: 676 (52%) Mixed: 494 (39%) Anaerobic: 123 (9%)	N/A	Aerobic: 199 (79%) Mixed: 43 (17%) Anaerobic: 11 (4%)	N/A
Intensity	Light: 768 (50%), Moderate: 653 (42%), Vigorous: 125 (8%)	N/A	Light: 579 (45%), Moderate: 602 (47%), Vigorous: 112 (8%)	N/A	Light: 189 (75%), Moderate: 51 (20%), Vigorous: 13 (5%)	N/A

*Note*: Data are presented as median [interquartile range] or *n* (%), as appropriate. T1DEXI participants represent the adult cohort, while T1DEXIP participants reflect the adolescent/paediatric cohort. Glycaemic metrics are derived from continuous glucose monitoring (CGM) data and include measures of average glucose, glycaemic variability (SD, CV, MAGE) and time in range (TIR). Physical activity (PA) characteristics reflect matched activity and non‐PA bout pairs, including starting glucose, insulin on board (IOB), bout duration and contextual variables such as PA type, intensity and time of day.

Abbreviation: CV, coefficient of variation.

### 3.2 Matching and PA Bout Inclusion

A comprehensive set of individual and event‐level variables was assessed for their influence on glucose responses to PA. Individual‐level variables included insulin modality, BMI and HbA1c. Event‐level variables included PA type, intensity, time of day, insulin dose, glucose rate of change, insulin on board (IOB), activity duration, starting glucose level and glucose coefficient of variation (CV) in the hour before activity. Following hierarchical modelling, the final matching algorithm retained four key variables: starting glucose, glucose rate of change, IOB and pre‐activity glucose CV. Of the 1944 eligible PA bouts, 398 were excluded due to insufficient match quality based on predefined thresholds. The remaining 1546 matched pairs were evaluated using SMDs, with SMDs <0.1 indicating successful covariate balance across matched variables.^19^

### 3.3 Characteristics at the Start of Physical Activity Bouts

Of the 1546 bouts, 1293 were from the T1DEXI cohort, while 253 were from the T1DEXIP cohort. Full pre‐PA characteristics are available in Table 1.

The overall median duration of PA bouts was 23 min (IQR: 20, 30), with a median of 25 min for T1DEXI participants and 20 min for those in the T1DEXIP group. Starting glucose level before activity for the total cohort was 11.7 mmol/L [10.7, 13.1], with it being 11.6 mmol/L [10.7, 12.9] for the T1DEXI group and 11.9 mmol/L [10.8, 14.0] for the T1DEXIP. Median IOB was comparable across groups, with T1DEXI having 0.01 U/kg (0.0, 0.03) and the T1DEXIP having 0.02 U/kg (0.0, 0.04) at PA onset.

### 3.4 Primary Outcome

#### 3.4.1 Effect of PA on Glucose Change

In the mixed‐effects model, after adjusting for relevant covariates and accounting for clustering by participant and matched bout, PA was associated with a significantly greater reduction in glucose compared with matched non‐PA periods (mean difference −1.9 mmol/L [95% CI −2.1 to −1.8]; *p* < 0.001). The mean glucose reduction following PA was −2.2 mmol/L (95% CI −2.4 to −1.9); *p* < 0.001, compared with −0.3 mmol/L (95% CI −0.4 to −0.2); *p* < 0.001following matched non‐PA periods. The glucose response for each PA bout, ordered by magnitude of drop and compared with its matched non‐activity period, is available in the Data (S3 and S4).

The model included random intercepts for both participants and matched PA/non‐PA periods, allowing each participant and each period to have its baseline level of the outcome. The random effects appropriately accounted for clustering by participant and the matched design, supporting the use of a multilevel structure in the model.

#### 3.4.2 Predictors of the differential glucose‐lowering effect of PA

**FIGURE 2:**
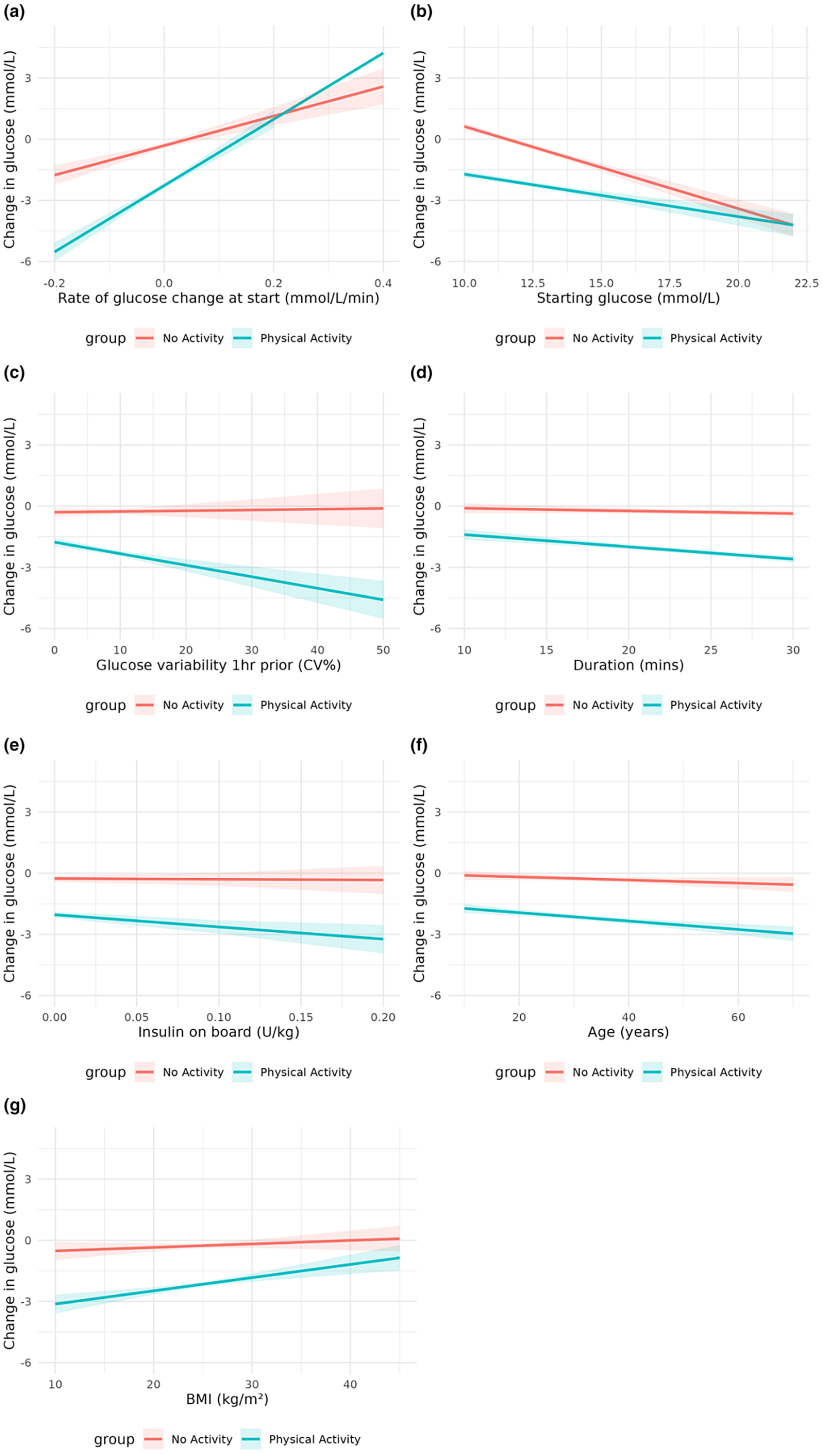
Individual‐ and event‐level moderators of the differential glucose drop between physical activity and non‐PA. Legend: This figure displays individual‐ and event‐level factors that influenced the difference in glucose change between physical activity (PA) and matched non‐PA periods: (a) starting glucose rate of change, (b) starting glucose level, (c) pre‐PA (1 h) glucose coefficient of variation (CV), (d) activity duration, (e) starting insulin on board (IOB), (f) age, (g) body mass index (BMI).

Further analyses examined factors modifying the effect of PA on glucose reduction. The glucose‐lowering effect of PA was greater in bouts with a faster rate of glucose decline at onset (per 0.1 mmol/L/min: −0.90 mmol/L [95% CI −1.19 to −0.61]; *p* < 0.001) (Figure 2a), lower starting glucose (per 1.0 mmol/L: −0.16 mmol/L [95% CI −0.23 to −0.09]; *p* < 0.001) (Figure 2b), higher pre‐PA CV (per 1%: −0.06 mmol/L [95% CI −0.09 to −0.03]; *p* < 0.001) (Figure 2c), longer duration (per minute: −0.05 mmol/L [95% CI −0.07 to −0.03]; *p* < 0.001) (Figure 2d) and higher IOB at onset (per 0.1 U/kg: −0.56 mmol/L [95% CI −1.09 to −0.04]; *p* = 0.035) (Figure 2e). Older age (per year: −0.01 mmol/L [95% CI −0.02 to 0.00]; *p* = 0.015) (Figure 2f) and lower BMI (per 1 kg/m^2^: −0.05 mmol/L [95% CI −0.09 to −0.01]; *p* = 0.012) were also associated with greater reductions. Full model results are provided in the Data (S3–S5).

### 3.5 Heatmap Decision Tool

**FIGURE 3:**
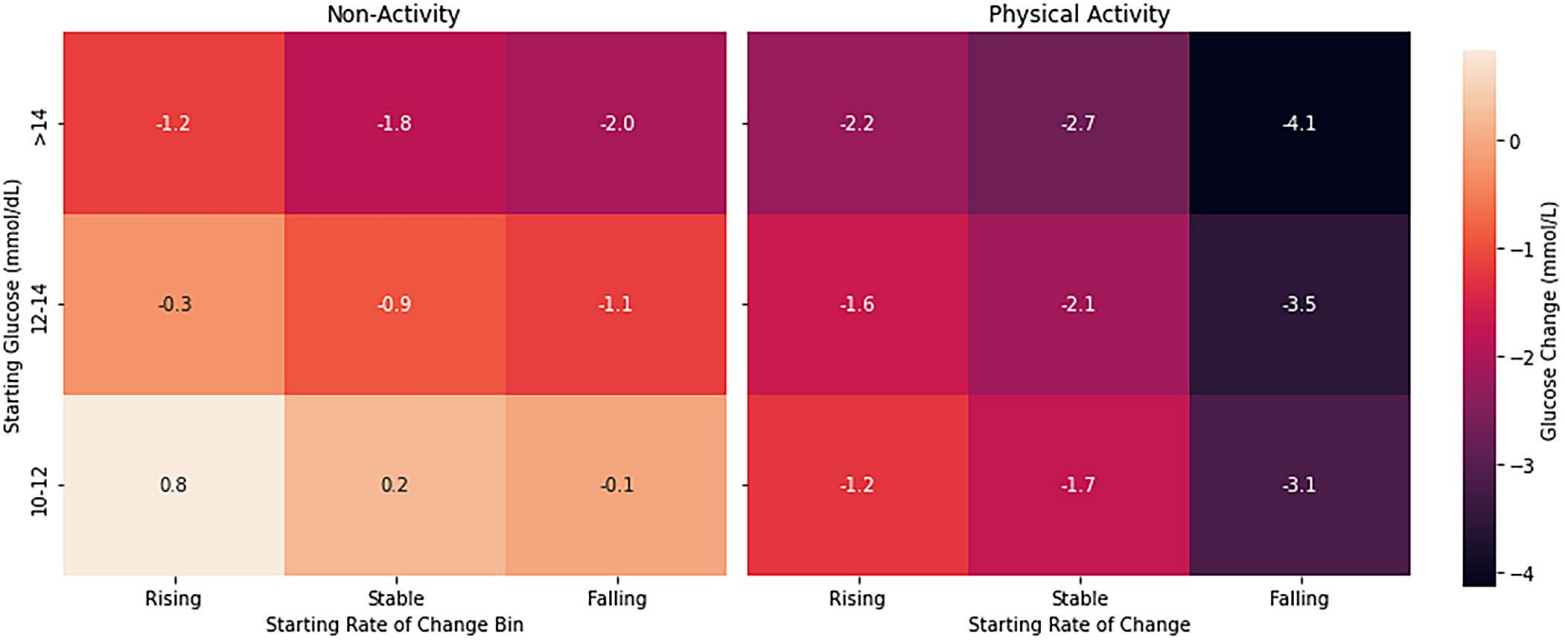
Predicted glucose trajectories based on physical activity status, starting glucose and glucose rate of change. This heatmap illustrates predicted glucose changes following a 23‐min (median) event with 20 min post‐event based on three key variables: physical activity status, starting glucose level and starting glucose rate of change. The figure shows how glucose responses differ when physical activity (PA) is undertaken versus not, across a range of starting glucose levels and trends. Warmer colours indicate greater predicted glucose reductions during PA.

The three strongest predictors of glucose change, PA status (defined as whether 10–30 min of activity was undertaken), starting glucose level and rate of glucose change at event onset, were used to model predicted glucose change (Figure 3, mg/dL version available in S5). Across all glucose levels and rates of change, PA events demonstrated greater glucose reductions. By integrating CGM‐derived glucose levels and rates of glucose change at the onset of physical activity, this tool provides a personalised estimate of the expected glucose response.

#### 3.5.1 Secondary Outcomes: Hypoglycaemia Risk

The absolute risk of hypoglycaemia during and up to 20 min after was 1.4% for PA bouts (22 out of 1546) and 0.1% for matched non‐PA periods (1 out of 1546). All of the hypoglycaemic episodes occurred in the adult T1DEXI dataset. Although the overall risk was low, PA was strongly associated with a higher likelihood of hypoglycaemia. In the fully adjusted generalised linear mixed model (binomial), PA was associated with a log‐odds increase of 4.48, corresponding to an odds ratio (OR) of 88 (95% CI: 88, 89, *p* < 0.001). Two additional factors were significantly associated with hypoglycaemia risk: starting glucose (log‐odds = −0.57, OR = 0.56) and event duration (log‐odds = 0.15, OR = 1.16). Other variables, including age, BMI, rate of glucose change at event onset and insulin delivery modality, were not significantly associated with risk. Full model results are provided in the Data (S6).

### 3.6 Sensitivity Analysis

To explore whether insulin or carbohydrate intake might have influenced glucose changes, a sensitivity analysis excluded PA bouts where insulin and/or carbohydrate were administered during the measurement period (PA onset to 20 min post‐activity). In the full dataset, 132 PA bouts included carbohydrate intake, 383 included insulin administration and 56 included both, yielding 303 unique bouts with either carbohydrate or insulin. For matched non‐PA periods, 80 included carbohydrate intake, 487 included insulin administration and 41 included both. These 303 PA bouts were excluded in the sensitivity analysis, leaving 1243 for analysis. Importantly, in line with best practice for causal inference,^23^ our models adjusted only for pre‐exposure confounders (starting glucose, glucose rate of change, insulin on board and pre‐PA coefficient of variation). Carbohydrate or insulin given after PA onset was treated as post‐exposure events and not included as covariates, to avoid introducing bias through adjustment for mediators or colliders. When these bouts were excluded, the average glucose drop during PA was slightly smaller (−2.0 mmol/L), and no hypoglycaemia bouts were observed (0 of 1243). This suggests that participants who consumed carbohydrates were likely reacting to a rapid glucose drop, and excluding these bouts could underestimate the true effectiveness and safety of PA for lowering glucose.

## 4 DISCUSSION

This study provides strong evidence that 10‐ to 30‐min bouts of PA significantly accelerate glucose lowering in people with type 1 diabetes compared to matched periods without PA. Using a within‐subject matched‐pairs design and 1546 free‐living PA bouts, we found that a median of 23 min of PA, initiated when glucose was above 10.0 mmol/L, lowered glucose by an average of 2.2 mmol/L compared with just 0.3 mmol/L during matched non‐PA periods. This represents an eightfold greater reduction attributable to PA, consistent across a diverse cohort of adolescents and adults using various insulin delivery methods and engaging in different types and intensities of activity. These findings demonstrate that even relatively brief periods of PA, using any form or intensity, can serve as an effective and practical strategy to rapidly lower elevated glucose levels.

These findings support the role of short bouts of physical activity (PA) as an adjunct to insulin for acute hyperglycaemia management, provided there is sufficient circulating insulin and no evidence of ketosis. Consistent with current guidance,^24^, ^25^ PA should be avoided when glucose is >15.0 mmol/L with blood ketones >1.5 mmol/L, as exercise under conditions of insulin deficiency may exacerbate hyperglycaemia and precipitate diabetic ketoacidosis. When glucose is elevated, ketone monitoring is recommended, particularly if symptoms suggest insulin deficiency; PA can be reconsidered once ketones are negative/trace and insulin has been administered to restore adequate circulating levels. Notably, prior work in the T1DEXI dataset showed that PA undertaken with insulin on board in the preceding 4 h was more likely to reverse hyperglycaemia,^8^ highlighting a potential synergistic effect between recent insulin administration and PA when ketosis is absent.

Causal inference methods are increasingly recognised as essential in diabetes research, particularly for generating real‐world evidence where even crossover study designs may not fully address confounding or reflect everyday conditions. Previous studies have shown that carefully matched observational data can mimic trial conditions to estimate treatment effects.^26^ For example, Deng et al.^27^ used this approach to closely replicate the GRADE trial, comparing glucose‐lowering therapies in people with type 2 diabetes using real‐world data. Our study builds on this framework by applying causal methods to PA and glucose control in people with type 1 diabetes. Using a within‐subject matched‐pairs design, each PA event was paired with a matched non‐PA period from the same individual. This allowed us to control for fixed factors like age, sex, insulin regimen and usual activity patterns, while also balancing key variables that change over time, such as starting glucose, glucose rate of change, CV and IOB.

This design closely mimics a ‘what‐if?’ comparison and strengthens causal inference by increasing confidence that the glucose‐lowering effects were due to PA itself, not other behavioural or metabolic factors. This approach marks a significant improvement over previous research using the T1DEXI and T1DEXIP datasets, which showed associations between PA and improved glucose but could not robustly infer causality.^8^

Our findings provide robust real‐world evidence that short bouts of PA, when used alongside insulin therapy, can rapidly lower glucose by ~2.0 mmol/L within 20 min, without increasing immediate hypoglycaemia risk. Although the odds ratio for hypoglycaemia during or within 20 min of PA was large, the absolute event rate was <2% and comparable with non‐PA periods. The high OR reflects a relative change from a near‐zero baseline rate in the non‐PA condition rather than a clinically significant increase in hypoglycaemia risk. The effect was consistent across different times of day, age groups and insulin delivery methods, underscoring its broad clinical relevance and supporting its potential as a foundation for future interventions and clinical guidance.

It is possible that part of the glucose‐lowering effect observed in the full dataset reflects a synergistic action of insulin and PA, particularly in cases where insulin was administered shortly before or at PA onset. This interpretation is supported by our prior analysis of the T1DEXI dataset, which showed that individuals with insulin on board (IOB) in the preceding 4 h were significantly more likely to reverse hyperglycaemia with activity.^8^ Together, these findings suggest that insulin may amplify the glucose‐lowering effects of PA when present, whereas in the absence of IOB, the effect is more likely to reflect insulin‐independent mechanisms of glucose uptake.

We identified starting glucose and glucose rate of change as the strongest predictors of glucose lowering with PA. A steeper downward glucose rate of change at activity onset led to a greater reduction, while a rising trend reduced the effect. Together, these variables explained much of the variation in glucose outcomes during both PA and matched non‐PA periods. This heatmap shows how starting glucose and rate of change, readily available from CGM, can guide personalised, real‐time decisions, refining the general finding that ~20 min of PA can lower glucose by ~2.0 mmol/L.

IOB was a key factor influencing the glucose‐lowering effect of PA. Higher IOB at activity onset significantly enhanced glucose reductions, consistent with physiological mechanisms whereby PA increases insulin sensitivity, accelerates subcutaneous insulin absorption and reduces renal insulin clearance.^5^, ^6^ Despite its clinical and research relevance, IOB remains challenging to quantify. In this study, it was estimated using a 4‐h linear decay model, which underestimates insulin action. Insulin can continue to act for five to 6 h,^28^ yet real‐world bolus calculators often assume shorter durations and overlook individual differences, limiting their utility for PA decisions.^29^

The absence of a significant effect of activity type or intensity is both surprising and encouraging. It suggests that individuals with type 1 diabetes can engage in a wide variety of activities and still achieve clinically meaningful glucose reductions. This flexibility may enhance adherence and allow for more personalised, lifestyle‐integrated approaches to glucose management through PA.

Personal factors also influenced the glucose‐lowering effect of PA. Participants with lower BMI had greater glucose reductions, likely due to higher baseline insulin sensitivity. This is consistent with previous findings from the T1DEXI and T1DEXIP cohorts.^11^, ^16^ Those with higher HbA1c levels showed smaller reductions, suggesting that chronic hyperglycaemia and insulin resistance may blunt the effectiveness of PA. Higher baseline fitness also appeared to enhance glucose response, which is supported by mechanistic studies showing that regular PA improves insulin action by increasing GLUT4 expression and reducing insulin resistance.^5^

This study has several important strengths. It is the first large‐scale analysis to use a within‐subject matched‐pairs causal design to evaluate the acute effect of PA on hyperglycaemia in people with type 1 diabetes using real‐world CGM data. Matching each PA bout to a non‐PA bout from the same individual controlled for fixed person‐level and key time‐varying factors, strengthening causal inference and providing a robust estimate of PA's independent effect on postprandial glycaemia. The analysis included over 1500 matched pairs across more than 400 participants, spanning a wide age range, insulin delivery methods, and types and intensities of PA.

Several limitations should be acknowledged. First, although matching reduces bias, residual confounding may remain, particularly from unmeasured factors such as sleep, stress or unrecorded food intake. Second, PA was self reported in the T1DEXIP cohort and device‐measured in the T1DEXI cohort, potentially introducing variability in event classification or intensity estimates. Third, IOB was estimated using a simplified 4‐h linear decay model that does not fully reflect rapid‐acting insulin pharmacodynamics. Fourth, the study focused on 10‐ to 30‐min PA and glucose effects up to 20 min post‐activity, limiting assessment of longer‐term outcomes like 2‐h AUC and delayed hypoglycaemia. We selected a 20‐min post‐PA observation window to capture the full acute glucose decline, as earlier work^8^ demonstrated that most of the effect is complete within this time frame. Extending the window would risk substantial data loss due to food intake soon after PA. Nonetheless, we acknowledge that assessing glucose trajectories over 1–2 h post‐PA would provide further insight into delayed hypoglycaemia risk, especially for prolonged aerobic activity with high IOB. Fifth, the small number of anaerobic and vigorous PA bouts limits the power to detect type‐specific glucose responses. While our analysis did not identify distinct effects from these bout types, the wide confidence intervals reflect reduced precision. Prior work using the full T1DEXI dataset (Pemberton et al., 2024) found that activity type was a weaker predictor of glucose change compared with IOB and starting glucose, suggesting that individualised physiological context may outweigh activity classification. Nonetheless, our proposed heat map should be validated in future datasets with broader representation of exercise intensities. Lastly, given the homogeneous cohort and varied PA modes and intensities, further research is needed across more diverse populations.

In summary, a 20‐min PA session lowered glucose by an average of ~2 mmol/L, which could be clinically translated into a simple ‘2‐every‐20’ rule. The strong influence of starting glucose and glucose rate of change supports the use of heatmaps and digital tools to personalise this strategy in education and clinical care.

## AUTHOR CONTRIBUTIONS

**John S. Pemberton:** Conceptualisation, background research and writing—original draft. **Catherine L. Russon:** Background research, formal analysis and writing—original draft. **Richard M. Pulsford:** Analysis and writing—review and editing. **Brad S. Metcalf:** Analysis and writing—review and editing. **Emma Cockcroft:** Writing—review and editing. **Michael J. Allen:** Analysis and writing—review and editing. **Anne M. Frohock:** Writing—review and editing. **Rob C. Andrews:** Supervision, writing—review and editing and intellectual revision.

## FUNDING INFORMATION

*Original Study Support*: This study was conducted as part of the T1DEXI Initiative, supported by the Leona M. and Harry B. Helmsley Charitable Trust. *Non‐Financial Contributions*: Verily Life Sciences (South San Francisco, CA) provided the Study Watch devices at no cost. Dexcom Inc. supplied continuous glucose monitoring systems at a discounted rate.

## CONFLICT OF INTEREST STATEMENT

John Pemberton: report being on the advisory board for Abbott and ROCHE and speaker fees from Abbott, Dexcom and Insulet in the last 3 years. Faculty member of exercise for type 1 diabetes. Catherine L. Russon: no conflicts. Richard M. Pulsford: no conflicts. Bradley S. Metcalf: no conflicts. Emma Cockroft: no conflicts. Michael Allen: no conflicts. Anne‐Marie Frohock: reports consultancy fees for Insulet and speaker fees from Dexcom and Insulet in the last 3 years. Faculty member of exercise for type 1 diabetes. Robert C. Andrews: reports research funding from Novo Nordisk Healthcare Organisation in the last 3 years, honoraria from Novo Nordisk, AstraZeneca and Eli Lilly for education talks on diet and exercise to health care professionals. Co‐founder of exercise for type 1 diabetes.

## GUARANTOR STATEMENT

Rob C. Andrews, Catherine L. Russon and John Pemberton are the guarantors of this work and, as such, had full access to all the data in the study and take responsibility for the integrity of the data and the accuracy of the data analysis.

## PRIOR PRESENTATION

Accepted for oral presentation at the European Association for the Study of Diabetes (EASD) Annual Meeting 2025, Vienna, Austria.

## Supporting information


Data S1.

## Data Availability

The data that support the findings of this study are available from the corresponding author upon reasonable request. All code is available at https://github.com/cafoala/glucoseLo‐stats.
