# Supplementary material for: Glucose‐lowering effects of physical activity in type 1 diabetes: A causal modelling and matched‐pair analysis approach
Source: Diabet Med. 2025 Oct 8;42(12):e70146. doi: 10.1111/dme.70146 (PMC12628732; doi:10.1111/dme.70146)
Supplement: Supplementary file 1 — Data S1. [file DME-42-e70146-s001.docx]

# Supplementary Data

**S1. Balance Diagnostics for Matched Physical Activity and Non-Activity Bouts.** *Standardised mean differences (SMDs) were used to assess balance between matched physical activity (PA) and non-PA bouts across key variables. An SMD <0.1 was considered indicative of good balance. Variables include demographic characteristics, clinical features, and bout-specific glucose and insulin measures. Balance diagnostics were conducted after matching to ensure comparability of activity and non-activity events.*

| **Variable** | **SMD** |
| --- | --- |
| IOB/kg | 0.090 |
| CV | 0.023 |
| Start glucose | 0.017 |
| Start rate of change | 0.007 |

**S2. Linear Mixed-Effects Model Predicting Change in Glucose Following Physical Activity Events.** *Results are from a linear mixed-effects model assessing the change in glucose (mg/dL) following physical activity (PA) bouts, with random intercepts for bout ID and participant ID. Fixed effects include demographic variables, bout characteristics, and their interactions with PA. All predictors were mean-centered but not standardised to preserve interpretability in original units. Estimated coefficients are reported with 95% confidence intervals*

| **Term** | **Estimate [95 % CI]** | **p-value** |
| --- | --- | --- |
| (Intercept) | −0.27 [−0.39, −0.15] | 1.34 × 10⁻⁵ |
| Exercise | −1.91 [−2.05, −1.77] | < 2 × 10⁻¹⁶ |
| Duration | −0.013 [−0.030, 0.004] | 0.127 |
| Start ROC | 7.25 [5.02, 9.47] | 1.95 × 10⁻¹⁰ |
| Age | −0.0076 [−0.016, 0.0006] | 0.089 |
| CV | 0.0038 [−0.018, 0.026] | 0.744 |
| BMI | 0.017 [−0.013, 0.048] | 0.265 |
| IOB (U kg⁻¹) | −0.34 [−4.25, 3.57] | 0.864 |
| Starting glucose | −0.40 [−0.46, −0.35] | < 2 × 10⁻¹⁶ |
| HbA1c | 0.031 [0.020, 0.042] | 5.97 × 10⁻⁸ |
| Exercise × Duration | −0.047 [−0.069, −0.025] | 4.07 × 10⁻⁵ |
| Exercise × Start ROC | 9.02 [6.02, 12.02] | 4.35 × 10⁻⁹ |
| Exercise × Age | −0.013 [−0.024, −0.002] | 0.015 |
| Exercise × CV | −0.060 [−0.091, −0.029] | 9.69 × 10⁻⁵ |
| Exercise × BMI | 0.048 [0.011, 0.085] | 0.0115 |
| Exercise × IOB (U kg⁻¹) | −5.64 [−10.89, −0.39] | 0.035 |
| Exercise × Glucose | 0.195 [0.123, 0.267] | 1.36 × 10⁻⁷ |

**S3. Random Effects from the Linear Mixed-Effects Model Predicting Glucose Change Following Physical Activity.** *This table presents the variance components and standard deviations for the random effects included in the mixed-effects model described in Table 1. Random intercepts were specified for both physical activity bout ID and participant ID to account for repeated measures within individuals and within activity events. The residual variance reflects unexplained variability after accounting for fixed and random effects.*

| **Group** | **Effect** | **Variance** | **Std. Dev.** |
| --- | --- | --- | --- |
| bout_id | Intercept | 3.469 | 1.863 |
| ID | Intercept | 119.067 | 10.912 |
| Residual | — | 1371.865 | 37.039 |

**S4. Mean ± SD Glucose Change per Participant (≥3 PA Events): Exercise vs Matched Non-Exercise Bouts*.*** *This figure displays the mean change in glucose (mg/dL) for participants with ≥3 physical activity (PA) bouts, comparing PA (red) to matched non-PA bouts (blue). Each point represents a participant’s average glucose change, with horizontal lines indicating ±1 standard deviation. Participants are ordered by the magnitude of their average glucose drop during exercise. This plot illustrates considerable inter-individual variability, with most participants experiencing a greater glucose-lowering effect during PA than in matched non-activity periods.*


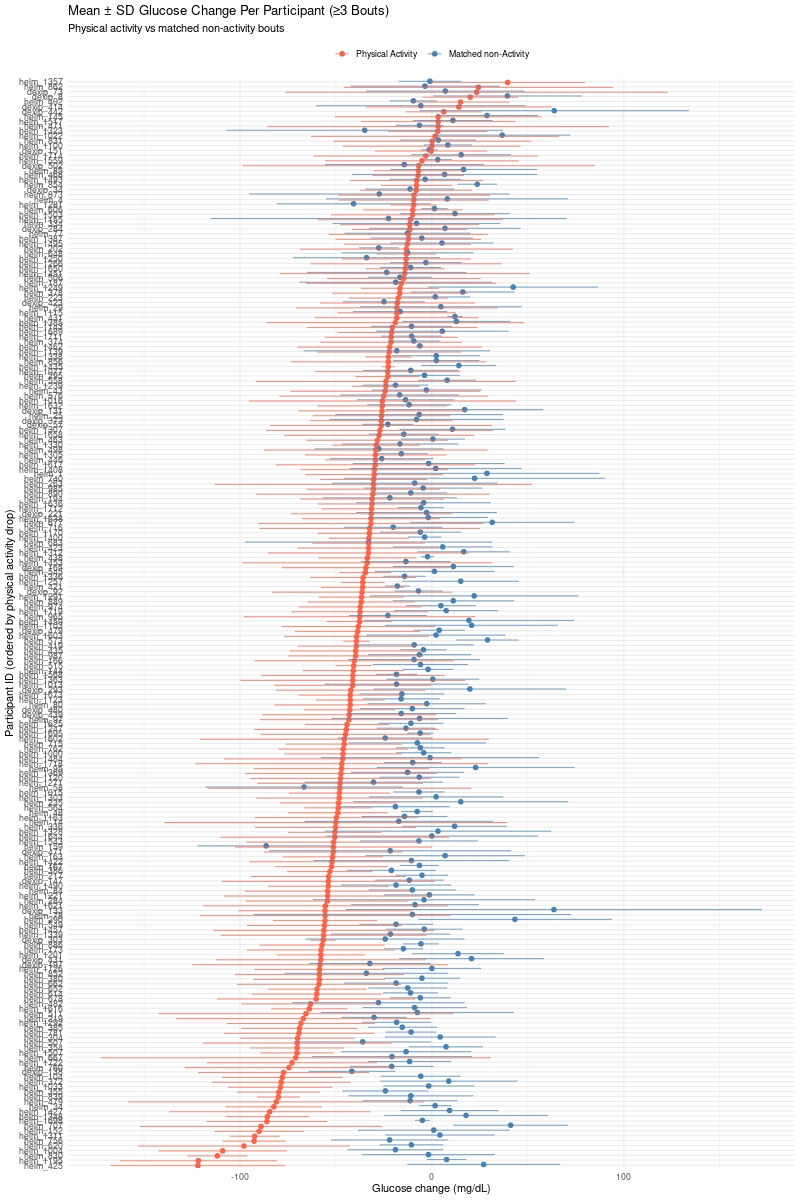


**S5.** Predicted Glucose Trajectories Based on Physical Activity Status, Starting Glucose, and Glucose Rate of Change


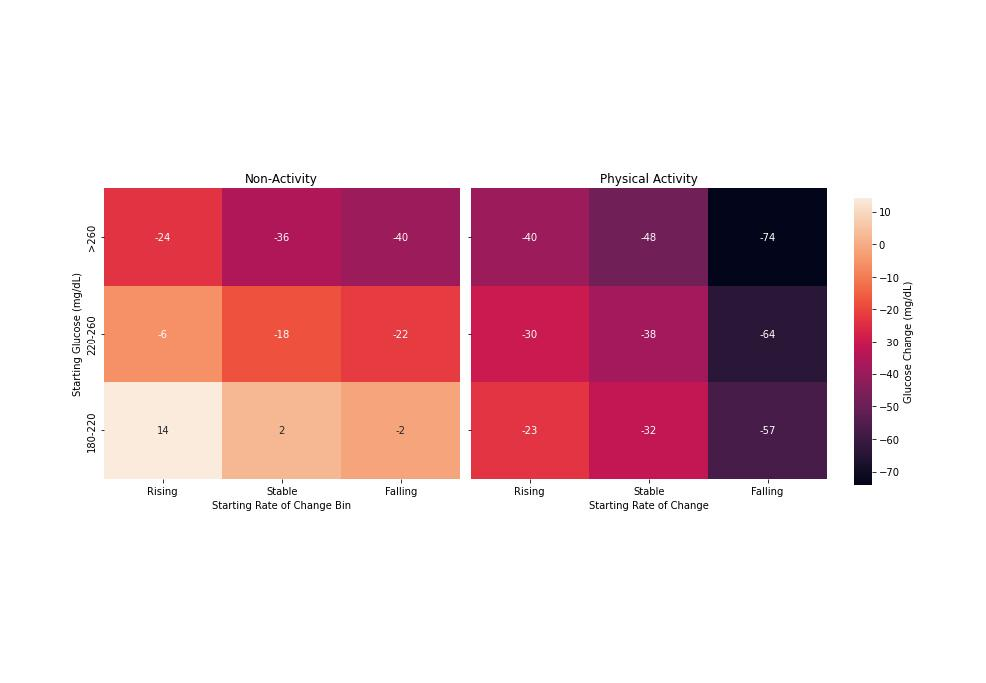


**Legend:** This heatmap illustrates predicted glucose changes following a 23-minute (median) event with 20-minutes post-event based on three key variables: physical activity status, starting glucose level, and starting glucose rate of change. The figure shows how glucose responses differ when physical activity (PA) is undertaken versus not, across a range of starting glucose levels and trends. Warmer colours indicate greater predicted glucose reductions during PA.

**S6. Summary of Variables Included in the Hypoglycemia Risk Model.** *This table provides a detailed overview of variables used in the hypoglycemia analysis pipeline. Each variable is listed alongside its type, unit (where applicable), and a brief description. Variables include participant demographics, bout characteristics, glycaemic metrics, and derived predictors (e.g. glucose rate of change, insulin on board). Variables were derived from CGM and pump data collected around physical activity events.*

| **Variable** | **Brief description** |
| --- | --- |
| form_of_exercise | Categorical label for whether the physical activity was aerobic, anaerobic or mixed |
| time_of_day | When the session began—typically coded as morning / afternoon / evening or a clock time |
| day_of_week | Day on which the session occurred (Monday … Sunday) |
| insulin_modality | Therapy type used for the session (e.g., MDI vs pump) |
| sex | Participant’s biological sex |
| food_pre_30 | Grams of carbohydrate eaten ≤ 30 min before exercise |
| food_pre_60 | Grams of carbohydrate eaten 30–60 min before exercise |
| iob_kg | Insulin on board per kilogram at the start of physical activity |
| ins_hrs | Number of hours since last insulin bolus |
| start_glc | Starting glucose at exercise onset (mmol L⁻¹) |
| start_roc | Glucose rate-of-change at onset (mmol L⁻¹ min⁻¹) |
| time_since_last_ins_dose | Minutes since last insulin bolus/basal adjustment |
| duration | Exercise bout length (minutes) |
| intensity | Intensity measure (e.g., %HRmax, METs, RPE score) |
| hba1c | Glycated haemoglobin—long-term glycaemic control (mmol/mol) |
| bmi | Body-mass index (kg m⁻²) |
| age | Participant age (years) |
| time_since_last_activity | Hours since the previous structured physical activity |
| Average glucose (mmol/L) | Mean glucose over the analysis window in the 1hr prior to exercise |
| SD (mmol/L) | Standard deviation of glucose values in the 1hr prior to exercise |
| CV (%) | Coefficient of variation of glucose (SD ÷ mean × 100) in the 1hr prior to exercise |
| AUC (mmol h L⁻¹) | Area under the glucose-time curve in the 1hr prior to exercise |
| LBGI | Low Blood Glucose Index—aggregate hypoglycaemia risk in the 1hr prior to exercise |
| HBGI | High Blood Glucose Index—aggregate hyperglycaemia risk in the 1hr prior to exercise |
| MAGE (mmol/L) | Mean Amplitude of Glycaemic Excursions in the 1hr prior to exercise |
| TIR normal (%) | % of time 3.9–10 mmol L⁻¹ (primary “time-in-range”) in the 1hr prior to exercise |
| TIR level 1 hypoglycemia (%) | % time 3.0–3.8 mmol L⁻¹ (“level 1” hypo) in the 1hr prior to exercise |
| TIR level 2 hypoglycemia (%) | % time < 3.0 mmol L⁻¹ (“level 2” hypo) in the 1hr prior to exercise |
| TIR level 1 hyperglycemia (%) | % time 10.0–13.9 mmol L⁻¹ (“level 1” hyper) in the 1hr prior to exercise |
| TIR level 2 hyperglycemia (%) | % time ≥ 14.0 mmol L⁻¹ (“level 2” hyper) in the 1hr prior to exercise |
| Total number hypoglycemic events | Count of all hypoglycaemic episodes in the 1hr prior to exercise |
| Number LV1 hypoglycemic events | Count of level 1 hypoglycaemic episodes in the 1hr prior to exercise |
| Number LV2 hypoglycemic events | Count of level 2 hypoglycaemic episodes in the 1hr prior to exercise |
| Number prolonged hypoglycemic events | Hypo events exceeding the set length threshold in the 1hr prior to exercise |
| Total number hyperglycemic events | Count of all hyperglycaemic episodes in the 1hr prior to exercise |
| Number LV1 hyperglycemic events | Count of level 1 hyperglycaemic episodes in the 1hr prior to exercise |
| Number LV2 hyperglycemic events | Count of level 2 hyperglycaemic episodes in the 1hr prior to exercise |
| Number prolonged hyperglycemic events | Hyper events exceeding the set length threshold in the 1hr prior to exercise |

**S7. STOBE Checklist.**

| STROBE Item | Description of How Item Was Met | Page(s) in Manuscript |
| --- | --- | --- |
| Title and Abstract | (a) The abstract clearly identifies this as a "within-subject matched-pairs causal design" study. (b) The abstract provides a balanced summary of the study’s aims, methods, results, and implications. | Abstract (p.1) |
| Background/Rationale | The introduction explains the burden of hyperglycemia in T1D and the limitations of insulin correction, establishing the need for alternative approaches such as physical activity. | Introduction (p.2) |
| Objectives | The study explicitly aims to evaluate the glucose-lowering effects of bouts of PA using a causal inference framework and matched control design. | Introduction (p.2) |
| Study Design | A within-subject matched-pairs observational study was used, applying causal inference techniques to CGM data. | Research Design and Methods (p.3) |
| Setting | The setting involved free-living environments using data from T1DEXI and T1DEXIP cohorts. Data were collected via CGM and accelerometer devices. No specific recruitment window is required due to the retrospective nature. | Research Design and Methods (p.3) |
| Participants | (a) Eligibility criteria: use of CGM, insulin, and accelerometer data with >70% completeness; PA initiated at glucose >180 mg/dL, n=482 (n=363 adults, n=119 adolescents) (b) Matching criteria: starting glucose, glucose rate of change, IOB, CV; n=1546, matched pairs. | Research Design and Methods (p.3-4) |
| Variables | Outcomes: change in glucose post-PA; exposures: PA vs. matched non-PA periods; confounders and modifiers: IOB, starting glucose, glucose rate of change, BMI, etc. | Research Design and Methods (p.4) |
| Data Sources/Measurement | T1DEXI and T1DEXIP cohorts; sensor glucose, insulin, and accelerometer data. Standardised methods across participants. | Research Design and Methods (p.4) |
| Bias | Within-subject design, outcome-oriented matching, and use of robust causal matching minimise confounding and selection bias. | Research Design and Methods (p.5) |
| Study Size | Initial PA bouts (n=1,943) were reduced to 1,546 matched pairs based on match quality criteria. | Research Design and Methods (p.5) |
| Quantitative Variables | All continuous variables (e.g., glucose, glucose rate of change, IOB) included in regression models; no arbitrary grouping. | Statistical Analysis (p.5-6) |
| Statistical Methods | (a) Linear/logistic mixed-effects models used to compare glucose change and hypoglycemia odds. (b) Subgroup analyses included moderators (age, BMI, etc.). (c) Missing data addressed through exclusion of poorly matched pairs. (d) Not applicable—study used observational datasets with complete follow-up periods. (e) Matching thresholds and variable weighting were sensitivity-tuned. | Statistical Analysis (p.6) |
| Participants (Results) | (a) 531 participants included, yielding 1,944 PA bouts. (b) 107 PA bouts excluded due to insufficient matching. (c) A flow diagram provided to show inclusions/exclusions | Results (p.7) |
| Descriptive Data | (a) Participant demographics, insulin modality, and glycemic metrics detailed in Table 1. (b) No missing data reported for key variables. (c) Follow-up time not relevant due to short-term outcome. | Table 1 & Results (p.8) |
| Outcome Data | Glucose changes and hypoglycemia outcomes are reported with means and confidence intervals. | Results (p.8-9) |
| Main Results | (a) Estimates presented with 95% CIs; adjusted for key covariates. (b) Continuous variables were retained as-is. (c) Absolute hypoglycemia risk (1.4%) reported alongside OR. | Results (p.9) |
| Other Analyses | Subgroup and moderator analyses performed and visualized using heatmaps and stratified effects. | Results (p.10) |
| Key Results | The glucose-lowering effect of PA was significant and robust across subgroups, summarized clearly in the discussion. | Discussion (p.11) |
| Limitations | Limitations include potential residual confounding, simplified IOB estimates, and absence of dietary data. | Discussion (p.12) |
| Interpretation | Results are cautiously interpreted, contextualized with prior literature, and practical implications are highlighted. | Discussion (p.12-13) |
| Generalisability | Real-world design, broad age range, and varied insulin modalities support generalisability. | Discussion (p.13) |
| Funding | Study supported by the Helmsley Charitable Trust. Devices provided at low/no cost by Verily and Dexcom. Funders had no role in analysis or reporting. | Acknowledgements (p.14) |
